# Career advancement of professional nurses at a regional hospital in Gauteng

**DOI:** 10.4102/curationis.v46i1.2453

**Published:** 2023-11-13

**Authors:** Crescelda L. van Biljoen-Mokhotla, Agnes Makhene

**Affiliations:** 1 Department of Nursing Health Sciences, Faculty of Nursing, University of Johannesburg, Johannesburg, South Africa

**Keywords:** perceptions, leadership, career advancement, professional nurses, mentorship, succession planning

## Abstract

**Background:**

Career advancement is of importance to professional nurses and a motivation for those who desire to occupy leadership positions. However, there were perceived barriers enunciated by participants, which were seen as contributory factors that hinder their progression in the institution.

**Objectives:**

The objective of the study was to explore and describe the perceptions of professional regarding their career advancement.

**Method:**

The study was conducted at a regional hospital in Gauteng. A qualitative, exploratory, descriptive design that is contextual was used. Non-probability purposive sampling method was used to draw the sample from a target population of professional nurses. Individual semi-structured interviews were conducted with 10 professional nurses to obtain in-depth information on their perceptions. Tesch’s method of qualitative data analysis was used. Lincoln and Guba’s strategies of credibility, dependability, confirmability and transferability were used to establish trustworthiness of the study. Ethical considerations were applied throughout the study.

**Results:**

A lack of recognition of postgraduate qualifications, no opportunities for self-identification of interest to lead, no mentoring processes for potential leader and succession planning and emotional experiences were barriers to career advancement. The researcher recommended that recognition of further qualifications, enhancement of self-identification for leadership roles, mentoring of young professionals for leadership and succession planning be considered to enhance career development of the professional nurses.

**Conclusion:**

The findings suggest that professional nurses encounter significant challenges to career advancement in this regional hospital.

**Contribution:**

Nurse managers to identify, mentor and support professional nurses for leadership positions in their career.

## Introduction

Career advancement is defined as the upward or parallel progression of individuals inside and outside the organisation (Moalusi & Jones 2019:1). It is not only a means of additional responsibilities nor financial gain for professional nurses, but it also goes together with their need to enhance their leadership ability and skills, to have a greater influence and success in health institutions. This boosts their morale and keeps them motivated (Avery, Westwood & Richardson 2022:406). In the South African Department of Health, there are leadership opportunities for professional nurses and these include being the chief executive officer of hospitals, leadership to serve on hospital boards, deputy manager in nursing, assistant manager, clinical heads and operational manager, to mention a few. However, the number of professional nurses in these leadership roles are few, because there are perceived barriers to career advancement hindering the progression of professional nurses into leadership positions.

The National Strategic Plan for Nurse Education, Training and Practice (2012/13–2016/17:29) reiterates that there has been a decrease in dedicated nursing leadership positions and the main obstacle is the lack of management capabilities in health delivery in South Africa. It is therefore very important to support, develop and effectively promote professionals into leadership to build the Department of Health’s ability to meet health service delivery demands, as many professional nurses in this regional hospital are nearing retirement age, which will leave the hospital with no leadership. This is also seen in the fact that several professional nurses in this health establishment have been in the same rank for over 5 years without moving up the nursing career ladder, which necessitated this study.

The significance of promotion and progression in the profession is of importance to professional nurses and it goes three ways. Firstly, it is undisputed that staff promotion motivates professional nurses to develop a sense of value. Secondly, following advancement, they show greater commitment and responsibility towards their work. Thirdly, the empowered professional nurses become more competent and effective and perform their duties at an increased level, which will impact positively on patient outcomes (Berg & Ruppert 2019:4).

## Methods

This study was conducted by means of a qualitative, explorative, descriptive and contextual research design approach. Qualitative research enabled the researcher to obtain in-depth information by exploring, describing and understanding the perceptions of professional nurses regarding career advancement. The study was approved by the Research Ethics Committee (REC-732-2020) and the Gauteng Department of Health, as well as the Ethics Committee of the regional public hospital. This study was undertaken in accordance with the ethical principles required for research practice involving humans, which include principles of autonomy, confidentiality, non-maleficence, beneficence and justice, as set out in Dhai and McQuoid-Mason (2011:166).

### Participants’ demographic information

The participants included 10 female professional nurses. Their ages ranged from 30 years to 55 years. Two of them were mixed-race people while eight were African. Years of experience ranged from 5 to 20 years and were all still in the professional nurse rank. Three had postgraduate qualifications, namely education, management and the other three were advanced midwives and one trauma qualified. The remaining three had basic nursing qualification.

### Setting and sample

The research was conducted at a regional hospital in Gauteng where the researcher intentionally selected the participants who wanted to be part of the study and have worked for longer than 5 years as professional nurses. This regional hospital has 22 wards consisting of medical, surgical, obstetrics and gynaecology, intensive care, operating theatres and trauma disciplines. The hospital gets patients from the surrounding communities and clinics.

In this study, purposive sampling was used where the researcher consciously selected participants to gain information as to how they perceive their career advancement. The inclusion criteria included being a professional nurse, having 5 or more years of experience, willingness to participate and being employed at the regional hospital. Professional nurses who had less than 5 years of experience and not willing to participate were excluded. This method was appropriate for the study as the selected participants gave rich and in-depth information that answered the research objectives. The researcher arranged a debriefing session with each participant to present the topic of the research study, its purpose and aims (Creswell & Poth 2018:158). The sample size was determined by data saturation, which was achieved at the 10th participant. The saturation of data was determined by repeatedly getting the same responses from the participants with no new data emerging from the 8th to the 10th participants. The participants were good informants and they were able to reflect on their perceptions and communicated effectively, which reached a point where no new information was obtained and redundancy was achieved (Polit & Beck 2017:497).

### Data collection

After obtaining permission to do the study, the researcher also requested to use the hospital boardroom when it was available. Professional nurses were invited by the researcher to a briefing session, which was held at the hospital boardroom. The purpose of the briefing session was to explain the study purpose and objectives to the potential participants who were also told that participation is voluntary. All ethical considerations that would be applied were also explained, after which willing participants signed consent facilitated by the researcher. The briefing sessions catered for a limited numbers of professional nurses at a time as we had to observe the COVID-19 protocols.

Data were collected by means of individual semi-structured interviews to get in-depth information on their perceptions. Interviews were conducted in the boardroom with adherence to COVID-19 regulations and the duration of the interviews was 45 min – 60 min. The interviews were conducted by the researcher who is a psychiatric nurse and has interviewing skills. The participants and interviewer wore face masks, kept 1.5 m apart from each other and used the hand sanitiser at the entrance door of the boardroom. Consent to use an audio recorder was obtained from the participants. The participants were asked two questions, namely what are the perceptions of professional nurses regarding their career advancement at a regional hospital in Gauteng and what can be done to enhance career advancement? The researcher used open-ended questions with the intention to elicit in-depth information from the participants (Nieswiadomy & Bailey 2017:212). The researcher listened attentively to participants’ responses and continued by asking follow-up questions and probing based on how participants responded. The researcher used communication strategies, namely questioning, probing, focusing, reflecting and summarising to elicit information from participants (Holloway & Galvin 2017:88). Non-verbal communication was observed by the researcher and recoded as field notes (Jacobsen 2021:415).

### Data analysis

Thematic coding of qualitative data analysis was employed, using Tesch’s method to ensure consistent, clear, focused, in-depth understanding, interpretation and integration of the collected data (Creswell 2013:184). This included organisation of data and the transcription of interviews. The audiotape recorded data were transcribed verbatim before being analysed according to Tesch’s coding method (Creswell 2013:184). Similar quotes were grouped together from emerging themes to obtain commonalities. The initial step of preparing the data for analysis was started, which was to transcribe all interviews, type the field notes, scan the material and create a file naming system for the data to be easily located. The interviews and field notes were transcribed for the researcher to become more aware of important concerns in the data. The researcher repeatedly read through the transcripts and listened to the audiotapes to confirm the written responses with the purpose to determine any significant phrases or sentences that emerged. Emerging themes were written on the margin of the transcript. The researcher read comprehensively through the transcripts as to obtain a sense of the whole, to interpret and elicit correct understanding of the data. The researcher continued to reflect on the meaning of the data, by also making notes in the margins of ideas that came to mind. An independent coder who held a doctoral degree and had expertise in qualitative data analysis was used to confirm the findings. A consensus meeting was held between the researcher and the independent coder to verify the accuracy of the analysed data.

Trustworthiness was achieved through adherence to the criteria of credibility was established through prolonged engagement, in that the researcher took the findings back to two of the participants to confirm the analysed data. The researcher established dependability through provision of detailed description of methods that were used in the study and careful documentation during the semi-structured interviews to create an audit trail. Confirmability was ensured through detailing all methods and data collected as well as using an independent coder to confirm data analysis. Detailed description of methods used in this study established transferability of the findings to other contexts and for potential researchers to use the findings in other settings (Lincoln & Guba 1985:166–289).

The principles of ethical consideration were applied in this study to ensure the protection of human rights, which are autonomy, confidentiality, beneficence, non-maleficence and justice. Participants signed informed consent as well as consent to audio-record the interviews and were informed that their names will not appear in any of the documents used in the study. The participants were each given a pseudonym during the interviews and were informed that they may terminate their participation at any stage of the research with no consequences to them. They were also ensured privacy in as far as the interviews were concerned and were made aware that all their information will be kept in a password-locked electronic file as well as a locked cupboard in the researcher’s office for hard copy documents. Furthermore, they were told the only persons who will have access to the research documents are the researcher and their supervisor.

### Ethical considerations

Ethical clearance to conduct this study was obtained from the University of Johannesburg, Faculty of Health Sciences Research Ethics Committee (No. REC-732-2020).

## Results

The study revealed that the participants had their own perception regarding their career advancement, evident by the perceived barrier of a lack of opportunity for self-identification of being interested in leadership roles (Table 1). The definition of leadership has changed over the years according to different schools of thought; however, it is considered important and a crucial part of the overall success and effectiveness of an institution and equally fundamental in nursing. Leadership is not a command-and-control process, but an approach where leaders are equipped with the necessary tools to inspire others to follow their example (eds. Fitzpatrick & Glazer 2013:262).

**TABLE 1:** Themes and related subthemes.

Main themes	Subthemes
Barriers to their career advancement	1.1 A lack of recognition of postgraduate nursing qualification 1.2 A lack of opportunities for self-identification as being interested in leadership roles 1.3 No mechanisms in place to provide early identification and mentoring of professional nurses with leadership potential 1.4 A lack of succession planning
Emotional experiences related to career advancement	2.1 Feeling of frustration, anger and demotivation

Note: Central theme: Negative perception of professional nurses regarding their career advancement.

Northouse (2021:6) defines leadership as a process whereby a single person influences a group of people to achieve mutual goals. Bans-Akutey (2021:1) states leadership involves motivating, inspiring and directing a team to attain set objectives. Ellis (2021:5) asserts that professional nurses provide leadership by being role models in the delivery of nursing care, and they are responsible and accountable for nursing care, delegation of duties and supervising the care rendered by others in the team. Rankin and McGuire (2015:2) concur that leadership is a vital skill necessary for effective patient care and effective ward management.

Spence Laschinger et al. (2013:219) report that in preparation for a leadership role of the participants, it is imperative that managers identify, understand and address interests and expectations of professional nurses. Managers must be aware of factors that influence the participants’ interest in leadership, which are their age, gender, probability of further education, extrinsic motivation, self-efficacy, intrinsic motivation, career motivation factors, career motivation satisfaction and professional commitment. In addition to this are leadership development opportunities, which consist of developmental experiences, access to a mentor, impact of formal mentoring; perceptions of manager’s role, supervisor resonating leadership, current work experiences and work engagement. Hassmiller and Pulcini (eds.) (2020:276) state that managers need to believe and work with these future leaders by providing them with opportunities to demonstrate their competency and skills, which will empower them to become leaders. In agreement, Lanzoni, Meirelles and Cummings (2016:7) posit that professional nurses are recognised as leaders by their natural characteristics and by their training. However, they feel unable to exercise or initiate their leadership role as they would want to.

Participants’ comments were as follows:

‘I want to be appointed in a leadership role and not be a so-called team leader only due to convenience.’ (Participant 5)

‘We are expected to do managers’ duties but they don’t acknowledge us as leaders. What part of this is fair?’ (Participant 6)

‘You must be very strong willed to say you are interested.’ (Participant 7)

Cummings et al. (2021:2–10) assert that professional nurses must understand that their leadership is important to providing quality care to patients. Cummings et al. (2021) further point out that the factors that contribute to their nursing leadership role are often characterised by leadership practices, behaviour, traits and characteristics, influences of context, practice settings and leader participation in educational activities to develop leadership capabilities and cultivate development of a new generation of nurse leaders. Grindel (2016:9) describes professional nurses as clinical experts who work directly with patients and have significant influence over patient care, patient satisfaction, patient safety and work within the multidisciplinary team, which makes them leaders.

Bimray and Jooste (2014:199) highlight that managers are needed, who are capable of supporting professional nurses to exercise leadership and also assisting them to develop professionally. Holroyd (2015:2) argues that leadership starts with self, meaning that one first needs to get to know oneself better in order to appreciate others and understand how this can impact your interactions with others. Reddy and Jooste (2015:474) concur that for professional nurses to exercise self-leadership, they will be required to have self-direction and be self-motivated in order to achieve their identified goals of being in a leadership role.

Participants further reported as follows:

‘Some professional nurses are not assertive enough to speak out and say, may I be given an opportunity lead and they end up disadvantaged.’ (Participant 3)

‘Hmm it is never received well when you try to do something that the operational managers feel it was supposed to be initiated or done by her. So, if you try to do something it gets crushed and you end up knowing your place, so now I just work and go home.’ (Participant 5)

Mansour et al. (2020:177) define assertiveness as an interpersonal skill, an honest expression of an individual’s needs, feelings and opinion without infringing on the rights of others. When the participants try to be innovative and initiate their interest in leadership for the benefit of the working environment, patient care and self-benefit, they are perceived as individuals who just want to express their self-worth and to be seen as more successful. This influences their intention to challenge managers and raise their concerns regarding interest in their leadership role. According to Azizi et al. (2019:2), the benefit of assertiveness is the development of leadership, increased job satisfaction, stress reduction in the workplace, and increased commitment and accountability in rendering patient care.

Morrow, Gustavson and Jones (2016:45) indicate that speaking up to a manager would require professional nurses to have the courage to challenge their managers, especially knowing that this can be difficult because of the way they have been raised and their culture to obey authority. Ames, Lee and Wazlawek (2017:1) assert that being assertive is a way to stand and speak up about their concerns without fear of victimisation or being undermined by their managers.

## Discussion

Overall, this study suggests that professional nurses desire greater opportunities for career advancement which above all includes to occupy leadership roles within the institution. However, the professional nurses confront barriers of career advancement of which the lack of self-identification of being interested in leadership role emerged as a clear frustration and demotivation for professional nurses. Contrary to the findings of this study, one could argue that the professional nurse’s interest in leadership role can be seen as an escape from the daily nursing workload, their undesirable work schedules, low salary and inability to be assertive enough to speak up and not having autonomy. It is also possible to argue that their ambition for leadership role is based on the escape to have a new position with higher status, salary increase and their desire to have a greater influence in the strategic planning and operations of the institution.

According to professional nurses participating in this study, the role of their working environment for career advancement was emphasised from different perspectives, which included support from managers through the creation of leadership development and awareness programmes and planning of role transition of professional nurses. These finding were consistent with the National Strategic Plan for Nurse Education, Training and Practice (2012/13–2016/17:47), which states that capacitation of professional nurses through appropriate leadership role awareness programmes equips nurse leaders to compete equitably with other disciplines at all levels of management within the institution and further suggests that leadership positions should be filled with leaders selected on merit who endeavour to build the institution’s ability to meet healthcare service delivery demands.

Literature review further indicates that managers have an important role in creating a healthy environment for professional nurses’ career advancement. Doria (2015:81) reports that a leadership development programme is a systematic process in which professional nurses get involved in formal management education, informal learning, and are exposed to an executive and mentoring programme. This is made possible when health institutions identify and develop high potential internal professional nurses by using leadership development programme, which as a result can improve role transition, decrease replacement costs and reduce manager turnover rate.

Campbell et al. (2017:291) suggest that the Robert Wood Johnson Foundation Nurse Faculty Scholar programme’s overall goal and objective of a leadership training programme in nursing is to produce well-informed, competent nurses who can sustain success in a leadership role and would be able to face challenges and complex demands within the institution. These leadership training programme focus areas are personal development, leadership skills for research and scholarship, management skills for academic leadership, leadership in health policy and translation of research to nursing practice, and skills for teaching excellence and leadership in nursing education.

Sonnino (2016:26) concurs that the benefits from a leadership training programme include personal growth, career advancement and the development of a special camaraderie, which encourages collaboration and synergy among colleagues in the institution. However, relying only on a leadership training programme to develop new and aspiring leaders has a risk for both the nurse and the institution, because not all leadership programmes address the different leadership competencies or key components that are covered by the more comprehensive curricula. These leadership programmes may not truly prepare the professional nurse for a leadership role; therefore, the professional nurse is required to participate in different training opportunities. The cost of training where resources are limited is also a risk, especially when the participant did not develop into a leader or secure a position where the new skills may be applied (Sonnino 2016:26).

## Recommendations

According to this study results and from literature, it is recommended that nurse leaders create awareness of leadership roles to show that they support professional nurses. In addition, it is essential to provide professional nurses with opportunities to assume leadership roles as they play an important role in the institution and they are interested, capable, prepared and have the necessary knowledge and nursing competencies to promote patient care and safety. Nurse leaders are to identify professional nurses who want leadership role and who have leadership qualities and potential, through career development programmes. The study’s findings indicate that the participants have a genuine sense of curiosity and interest to pursue leadership positions. Therefore, it is important for managers to create a working environment that supports professional nurses’ engagement in leadership role, which will also make them committed to the institution. Ekström and Idvall (2015:72) emphasise leadership as one of the areas of expertise within the scope of professional nurses’ work. However, leading seems to be difficult for them, especially when they are not empowered by managers to lead teams in the nursing units, be innovative or initiate changes in the departments. Beauvais (ed. 2022:10) states that leadership is not only about a position held but also about knowledge processes, self-awareness and relationships with others. Turale and Kunaviktikul (2019:303) indicate that the International Council of Nurses and the World Health Organization have that indicated there is a need for nurse leadership and therefore nurses should move with the times and get more involved in leadership and development of policies.

## Conclusion

It is the researcher’s opinion that postgraduate qualifications should be recognised, and potential leadership interest needs to be nurtured. Young enthusiastic professional nurses should be mentored by senior nurses to assist them in building their leadership skills. They must be allowed to ‘shadow’ management to enable them to see first-hand and learn what nursing leaders do on a daily basis within the hospital. Career planning for them will prevent leaving a vacuum when leaders retire.

### Limitations

The study was conducted in one regional hospital in Gauteng. Different perceptions may have been described by professional nurses in other regional hospitals, which might impact the transferability of the findings. The interviews were conducted during the lunch times of the participants and they may have been overwhelmed by the workload in the department and unable to give adequate feedback. Some of the participants withdrew from the study because of fear of victimisation although the researcher gave extensive information regarding the research study, implications and confidentiality thereof.
